# The efficacy and safety of granulocyte colony-stimulating factor in the treatment of acute-on-chronic liver failure: A systematic review and meta-analysis

**DOI:** 10.1371/journal.pone.0294818

**Published:** 2023-11-30

**Authors:** Bo Qiu, Jia Xu Liang, Manuel Romero Gómez

**Affiliations:** 1 Department of Gastroenterology, The Second Affiliated Hospital of Hainan Medical University, HaiNan, China; 2 Department of Diagnostic Radiology, People’s Hospital of Zhengzhou, Zhengzhou, China; 3 Department of Digestive, Hospital Universitario de Virgen Rocio, Seville, Spain; Institute for Clinical and Experimental Medicine, CZECH REPUBLIC

## Abstract

**Background and objectives:**

The safety and efficacy of granulocyte-colony stimulating factor (G-CSF) for the treatment of acute-on-chronic liver failure (ACLF) remain controversial. This meta-analysis aimed to evaluate the effectiveness and safety of G-CSF in treating ACLF.

**Methods:**

The estimated pooled risk ratio (RR) and 95% confidence interval (CI) assessed the treatment effects of G-CSF. Mean differences (MD) and 95% confidence intervals were used to analyze continuous data. Heterogeneity was explored by sensitivity analysis. Potential publication bias was assessed using Egger’s test.

**Results:**

Ten studies, comprising a total of 603 participants, were included in the analysis. The G-CSF group showed significantly lower MELD scores at 7-day (MD = -2.39, 95%CI: -3.95 to -0.82), CTP scores at 7-day (MD = -0.77, 95%CI: -1.41 to -0.14), and MELD scores at 30-day (MD = -3.01, 95%CI: -5.36 to -0.67) compared to the control group. Furthermore, the G-CSF group was associated with a reduced risk of sepsis (RR = 0.53, 95%CI: 0.35–0.80). The 30-day survival (RR = 1.26, 95%CI:1.10–1.43), 60-day survival (RR = 1.47, 95%CI:1.17–1.84, and 90-day survival (RR = 1.73, 95%CI: 1.27–2.35) of patients with ACLF treated with G-CSF were significantly higher than those of the control group.

**Conclusions:**

Our meta-analysis suggests that G-CSF therapy may be a promising treatment for ACLF, with significant improvements in liver function and survival rates, however, further studies are needed to verify this conclusion.

## Introduction

Acute-on-chronic liver failure (ACLF) is a severe and life-threatening condition characterized by an acute deterioration of liver function in patients with pre-existing chronic liver disease [1]. ACLF can result from various etiologies, including viral hepatitis, alcohol-related liver disease, non-alcoholic fatty liver disease, and autoimmune liver disease [2]. It is characterized by severe systemic inflammation, organ failure, and poor prognosis [3,4]. The mortality exceeded 30% at 28 days and 63% at 90 days [5,6]. The only recommended treatment option is liver transplantation, but the scarcity and high cost of donor organs limit its use.

To overcome the challenges of treating advanced liver disease, researchers have proposed alternative methods such as bone marrow stem cell (BMSC) therapy and mobilization of granulocyte colony-stimulating factor (G-CSF). BMSCs are an important source of liver stem cells that possess the ability to differentiate into multiple cell types and self-renew. Studies have demonstrated that BMSCs can promote hepatocyte proliferation and liver regeneration, as well as regulate immune function [7,8]. G-CSF mobilizes hematopoietic stem cells in patients with advanced liver disease. It improves liver function and survival in ACLF patients. Compared to bone marrow stem cell transplantation, mobilization of autologous bone marrow stem cells to the damaged area of the liver is a simple, safe, effective, and non-invasive procedure that avoids the immune response associated with stem cell transplantation. This approach does not require stem cell extraction and expansion and has promising applications. G-CSF mobilizes hematopoietic stem cells and has been shown to improve liver function and increase hepatocyte regeneration in both animal models and clinical studies [9–11].

However, the effectiveness and safety of G-CSF treatment in ACLF are still being debated. A meta-analysis suggested that G-CSF treatment may only reduce short-term overall mortality but not mortality secondary to gastrointestinal bleeding [12]. Another meta-analysis showed no significant beneficial effects of G-CSF in ACLF [13]. Moreover, some studies had small sample sizes and insufficient statistical power to predict the outcomes of a comprehensive study [14,15]. Thus, the efficacy and safety of G-CSF in liver failure remain controversial. To address this issue, we conducted a large sample analysis in this meta-analysis to evaluate the safety and effectiveness of G-CSF in the treatment of ACLF.

## Method

### Literature search strategy

Two independent investigators (B. Q and JX. L) conducted a comprehensive search of the PubMed, Cochrane Library, and Embase to identify relevant studies published up to March 2023. The search strategy involved using MeSH terms and keywords, including (“granulocyte-colony stimulating factor” or “G-CSF”) and (“acute-on-chronic liver failure” or “ACLF" or “liver failure” or “Hepatic failure” or “severe hepatitis” or “Fulminant hepatitis”). No restrictions were placed on article type or additional filters during the search. A manual search was also conducted by reviewing the references of original articles and relevant review articles. The results were collected independently by the two investigators.

### Inclusion and exclusion criteria

Studies were considered suitable for this meta-analysis if they met the following inclusion criteria: (1) Randomized clinical trials (RCT); (2) The experimental group received G-CSF therapy while the control group received conventional treatment; (3) Availability of clinical outcomes. Exclusion criteria were (1) Non-therapeutic use of G-CSF; (2) Animal studies; (3) Non-randomized controlled studies.

### Data extraction

Two reviewers (B. Q and JX. L) independently extracted uniformly reported data elements for each study meeting the inclusion criteria, which were then cross-verified by a third reviewer (Romero Gomez M). In cases where the same population was reported in multiple articles, only the most informative or complete study was retained to prevent duplication. The following information was extracted from each study: first author, year of publication, country, sample size, etiology, route and dosage of G-CSF administration, survival rates, and complications. The Cochrane risk of bias tool was used to assess each trial, with standard criteria including the following domains: random sequence generation, allocation concealment, blinding of participants and personnel, blinding of outcome assessment, incomplete outcome data, selective reporting, and other biases [16].

### Statistical analysis

Pooled risk ratios (RR) and their corresponding 95% confidence intervals (CIs) were used to evaluate the therapeutic effect of G-CSF. Mean differences (MD) and 95% confidence intervals were used for continuous data analysis.

Heterogeneity between trials was quantified using the I^2^ test, with I^2^ ≥ 50% indicating significant heterogeneity. A fixed-effects model was used when heterogeneity was not significant. Obvious clinical heterogeneity was evaluated by removing a single study and repeating the meta-analysis [17].

We evaluated potential publication bias using Egger’s test, with a P value greater than 0.05 indicating no publication bias. All statistical analyses were performed using RevMan 5.4 (The Cochrane Collaboration, Oxford, UK) and Stata version 15 (Stata Corp, College Station, Texas, USA).

## Results

### Characteristics of the included studies

**Fig 1:**
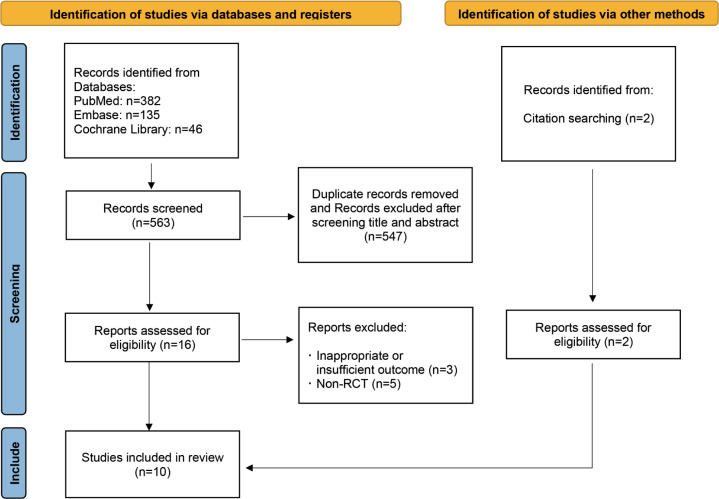
Study identification and selection flowchart.

**Fig 2:**
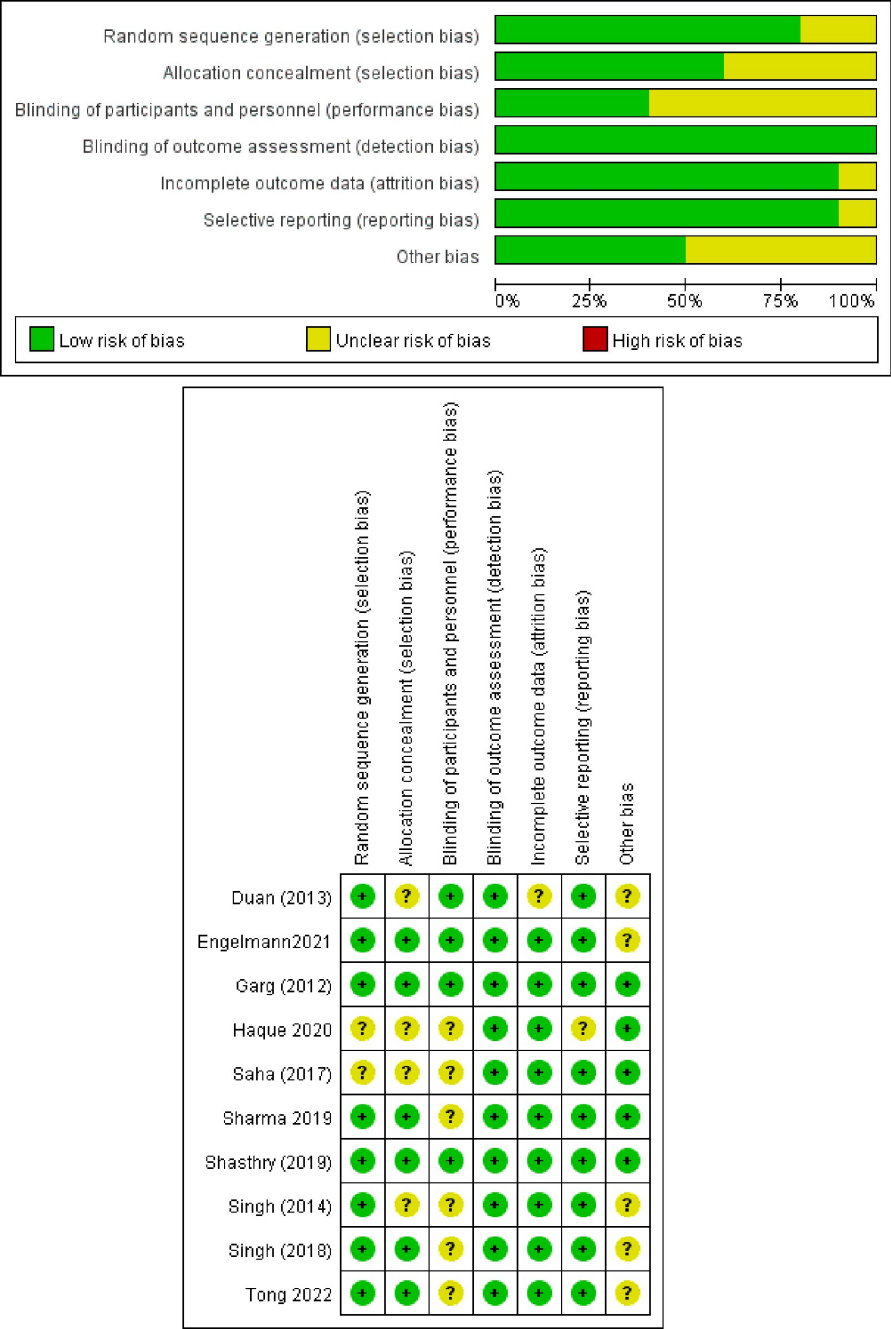
a. Risk of bias graph; b. Risk of bias summary.

Out of the 563 articles identified through searches of the Cochrane Library, PubMed, Embase, and references, 10 studies including a total of 603 participants were ultimately included in our meta-analysis [10,11,14,15,18–23]. A flowchart detailing the study identification and selection process is presented in Fig 1. Of the 10 studies analyzed, five were conducted in India [11,15,20–22], two in China [10,23], two in Bangladesh [14,19], and one in Germany [18]. The main characteristics of each study are summarized in Table 1. Prior to data analysis and synthesis, the quality of the studies was evaluated using the Cochrane risk of bias tool, as shown in Fig 2.

**Table 1:** Study characteristics.

Author (year)	Country	Sample size	Etiology	Drug route/dose	CTP score at baseline	MELD score at baseline	Survival (%) at 30 days	Survival (%) at 60 days	Survival (%) at 90 days	Complications
Duan (2013) [10]	China	55 G-CSF:27 Control:28	Hepatitis B	5 μg/kg/day subcutaneously for 6 consecutive days	G-CSF: 12.17 ± 1.47 Control: 12.25 ± 1.29	G-CSF: 25.11 ± 3.30 Control: 26.30 ± 4.12	————	————	G-CSF: 13 (48.1%) Control: 6 (21.4%)	Hepatorenal syndrome, hepatic encephalopathy, sepsis, hemorrhages
Engelmann (2021) [18]	Germany	176 G-CSF:88 Control:88	SAH	5 μg/kg/day subcutaneously for 5 consecutive days followed by every third day until 1 month	————	G-CSF: 24.4 ± 6.3 Control: 24.5 ± 6.1	G-CSF: 42 (47.7%) Control: 43 (48.9%)	G-CSF: 29 (33.0%) Control: 31(35.2%)	G-CSF: 27 (30.7%) Control: 26 (29.5%)	Hemorrhages, sepsis, organ failures
Garg (2012) [11]	India	47 G-CSF:23 Control:24	Mixed*	5 μg/kg/day subcutaneously for 5 consecutive days followed by every third day until 1 month	G-CSF: 12 (11–14) Control: 12 (10–14)	G-CSF: 29 (21–40) Control: 31.5 (20–40)	G-CSF: 18 (78.3%) Control: 11 (45.8%)	G-CSF: 16 (69%) Control: 7 (29%)	————	Hepatorenal syndrome, hepatic encephalopathy, sepsis, organ failures
Haque (2020) [19]	Bangladesh	39 G-CSF:22 Control:17	Mixed	5 μg/kg/day subcutaneously for 6 consecutive days	G-CSF: 11.77 ± 1.11 Control: 11.65 ± 1.36	G-CSF: 27.64 ± 4.60 Control: 29.47 ± 5.53	G-CSF: 14 (63.6%) Control: 10 (58.8%)	G-CSF: 11 (50%) Control: 5 (29.4%)	G-CSF: 8 (36.4%) Control: 5 (29.4%)	Hepatorenal syndrome, hemorrhages, hepatic encephalopathy
Saha (2017) [14]	Bangladesh	32 G-CSF:16 Control:16	Mixed	5 μg/kg/day subcutaneously for 6 consecutive days	G-CSF: 12 (10–13) Control: 12 (10–14)	G-CSF: 24.5 (21–32) Control: 25.5 (21–35)	G-CSF: 14 (87.5%) Control: 13 (81.3%)	G-CSF: 14 (87.5%) Control: 13 (81.3%)	G-CSF: 14 (87.5%) Control: 8 (50%)	Hepatorenal syndrome, electrolytic imbalance
Sharma (2019) [20]	India	31 G-CSF:15 Control:16	Mixed	5 μg/kg/day subcutaneously for 5 consecutive days	G-CSF: 12 ± 1.4 Control: 12.75 ± 0.85	————	G-CSF: 10 (66.7%) Control: 6 (37.5%)	G-CSF: 8 (53.3%) Control: 6 (37.5%)	————	None
Shasthry (2019) [15]	India	28 G-CSF:14 Control:14	SAH	5 μg/kg/day to a maximum of 300 μg per day for 5 doses followed by every third day until 4 weeks	G-CSF: 10.9 ± 1.3 Control: 10.7 ± 1.2	G-CSF: 24.6 ± 3.9 Control: 27.6 ± 4.4	G-CSF:11 (78.6%) Control: 10 (71.4%)	G-CSF: 10 (71.4%) Control: 5 (35.7%)	G-CSF: 9 (64.3%) Control: 4 (28.6%)	Hemorrhages, sepsis
Singh (2014) [21]	India	46 G-CSF:23 Control:23	SAH	5 μg/kg subcutaneously every 12 h for 5 consecutive days	G-CSF: 12 Control: 12	G-CSF: 27 Control: 30	G-CSF: 18 (78.3%) Control: 10 (43.5%)	G-CSF:18 (78.3%) Control: 9 (39.1%)	G-CSF: 18 (78.3%) Control: 5 (21.7%)	Hepatorenal syndrome, hemorrhages, hepatic encephalopathy
Singh (2018) [22]	India,	38 G-CSF:18 Control:20	SAH	5 μg/kg subcutaneously every 12 h for 5 consecutive days	G-CSF: 11 (8–13) Control: 10 (8–14)	G-CSF: 26 (19–37) Control: 27.5 (19–41)	G-CSF: 16 (88.9%) Control: 11 (55%)	G-CSF: 16 (88.9%) Control: 8 (40%)	G-CSF: 16 (88.9%) Control: 6 (30%)	Sepsis, hemorrhages, organ failures
Tong (2022) [23]	China	111 G-CSF:54 Control:57	Hepatitis B	5 μg/kg/day subcutaneously for 6 consecutive days	————	G-CSF: 22.8 (20.7–26.0) Control: 24.1 (21.6–27.1)	G-CSF: 46 (85.2%) Control: 38 (66.7%)	G-CSF: 39 (72.2%) Control: 31 (54.4%)	G-CSF: 36 (66.7%) Control: 30 (52.6%)	Sepsis, hepatic encephalopathy, acute kidney injury

NOTE. Data are presented as mean ± SD, n (%), or median (range).

Mixed

*: Viral hepatitis and alcoholic hepatitis related acute and chronic liver failure; SAH, severe alcoholic hepatitis; G-CSF, granulocyte colony-stimulating factor.

### Changes in liver function after G-CSF therapy

#### MELD and CTP score

**Fig 3:**
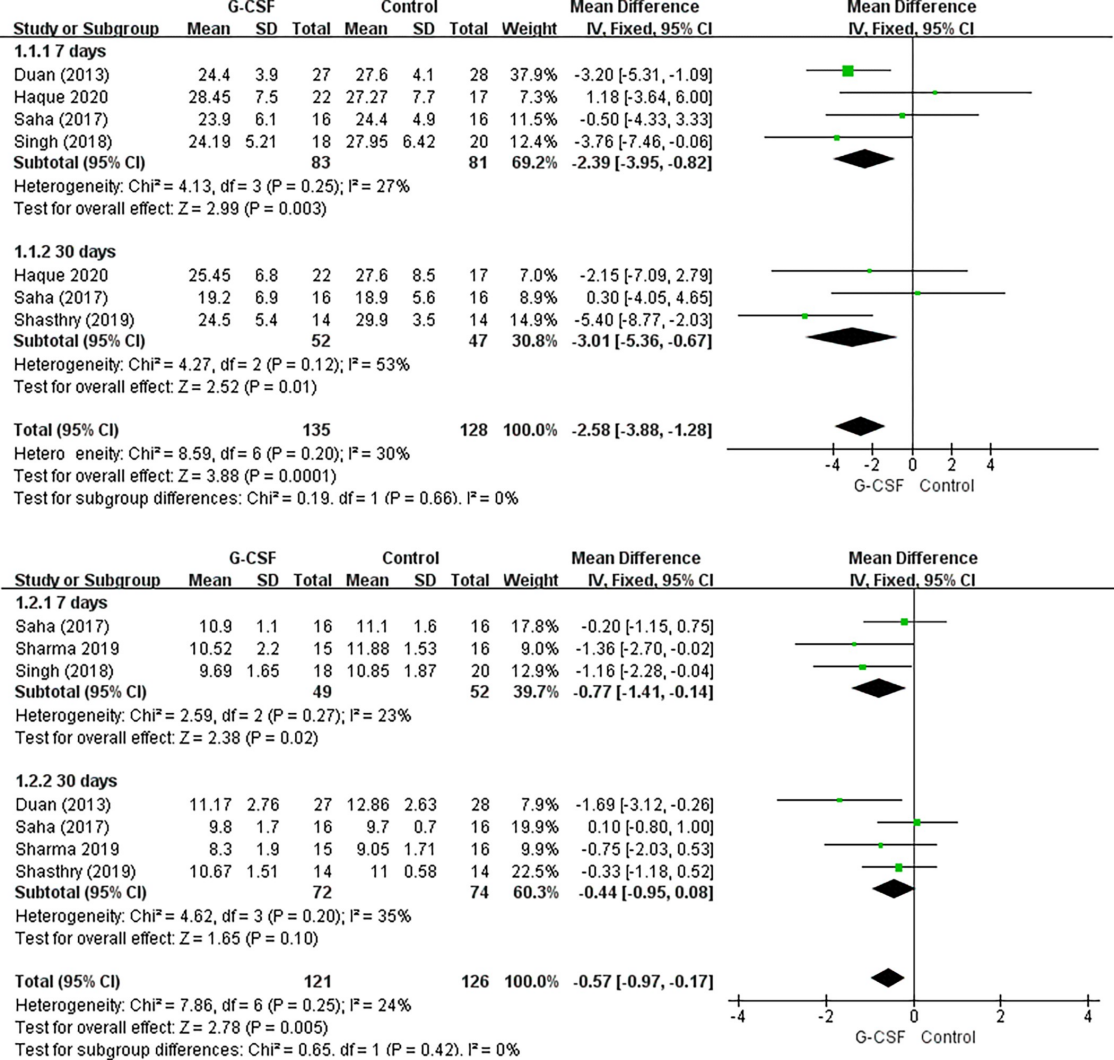
Meta-analysis of MELD and CTP scores between G-CSF and control therapy. a). MELD scores, b) CTP scores.

Six studies provided detailed post-treatment scores for the Model for End-Stage Liver Disease (MELD) and Child-Turcotte-Pugh (CTP) [10,14,15,19,20,22]. The results of the meta-analysis indicate that, compared to the control group, the experimental group showed a significant improvement in MELD scores (MD = -2.39, 95%CI: -3.95 to -0.82, P<0.05) and CTP scores (MD = -0.77, 95%CI: -1.41 to -0.14, P<0.05) after 7 days of treatment, and a significant improvement in 30-day post-treatment MELD score (MD = -3.01, 95%CI: -5.36 to -0.67, P<0.05). However, there was no significant difference in the 30-day post-treatment CTP score between the control and experimental groups (Fig 3A and 3B).

#### Laboratory results

Three studies that compared G-CSF with control therapy provided data on liver function parameters [15,21,22]. The meta-analysis results showed that G-CSF treatment did not result in a significant improvement in serum albumin (MD = 0.07, 95%CI: -0.16 to 0.31, P = 0.54, I^2^ = 0%) and total bilirubin (MD = -1.53, 95%CI: -5.47 to 2.40, P = 0.44, I^2^ = 0%), but international normalized ratio (INR) was slightly improved (MD = -0.25, 95%CI: -0.47 to -0.04, P<0.05, I^2^ = 12%) within one month.

### Prevention of serious complications and improvement of survival

#### Survival rates

**Fig 4:**
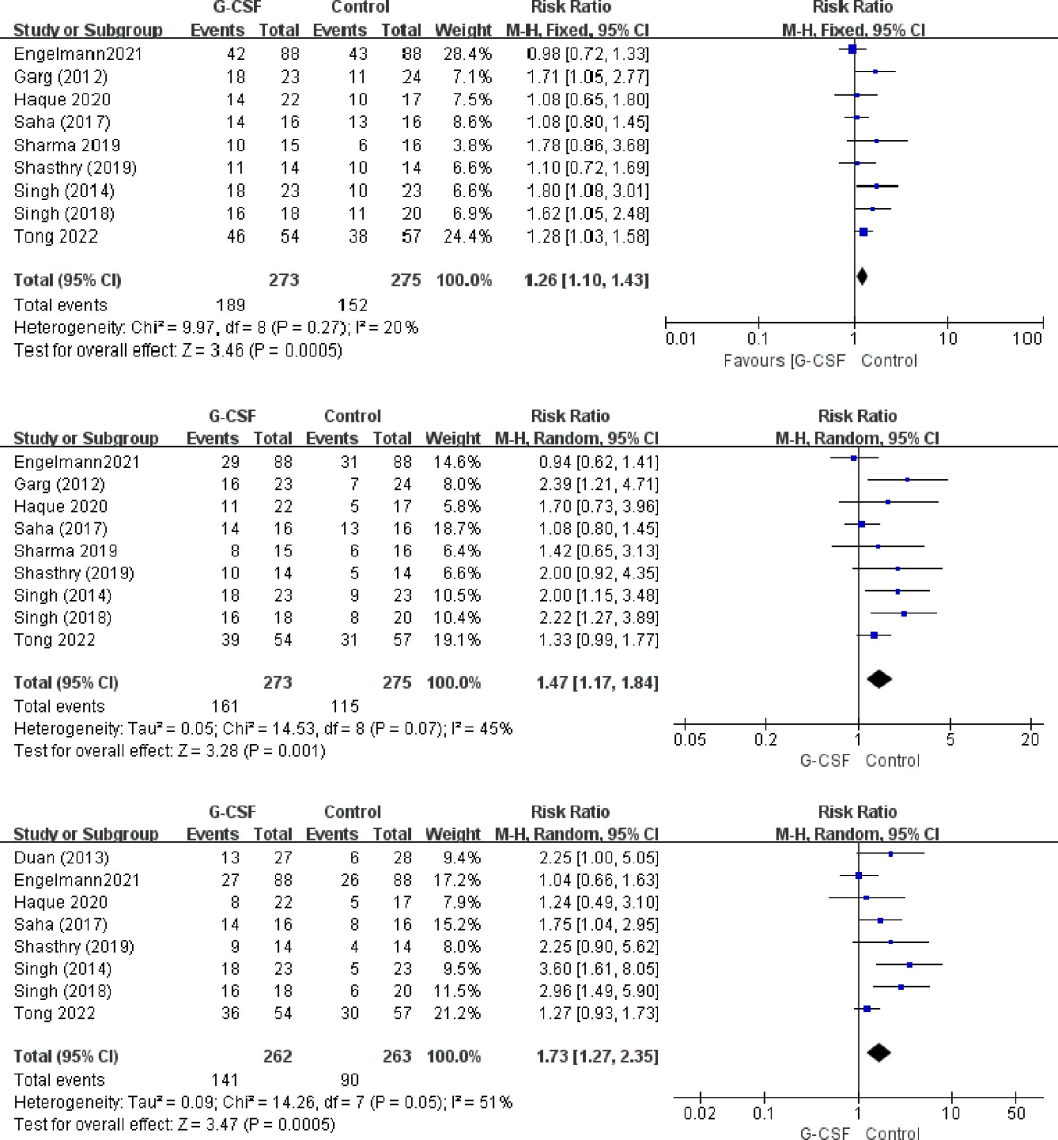
Survival rate between patients with ACLF, G-CSF groups vs. control group. a). 30-day, b) 60-day, c) 90-day.

The intervention methods used in the ten eligible studies were roughly commensurate, but there was heterogeneity between the articles (P = 0.05). Therefore, a random effects model was used to evaluate the 90-day survival rates. The results indicate that G-CSF therapy was associated with a reduced risk of death for patients with ACLF, with pooled RR (95% CI, P) values of 1.26 (1.10–1.43, P<0.05) for 30-day survival, 1.47 (1.17–1.84, P<0.05) for 60-day survival, and 1.73 (1.27–2.35, P<0.05) for 90-day survival (Fig 4A–4C).

We conducted a subgroup analysis to compare the effect of different doses (5 μg/kg/day vs. 10 μg/kg/day) of G-CSF in treating ACLF. The findings demonstrated that both doses improved the survival rates of ACLF patients at 30, 60, and 90 days (Table 2).

**Table 2:** Subgroup analysis based on dose for assessment of survival rate.

Category	Subgroups	RR	95%Cl	P	I^2^
30-day survival rate	5 μg/kg/day	1.19	1.03–1.36	<0.05	0%
	10 μg/kg/day	1.71	1.22–2.38	<0.05	0%
60-day survival rate	5 μg/kg/day	1.31	1.09–1.57	<0.05	31%
	10 μg/kg/day	2.10	1.42–3.12	<0.05	0%
90-day survival rate	5 μg/kg/day	1.37	1.11–1.70	<0.05	3%
	10 μg/kg/day	3.22	1.91–5.43	<0.05	0%

### Development of complications and analysis of the cause of death

Sepsis was reported in 6 studies involving 554 ACLF patients, 224 were in the G-CSF treatment group and 230 were in the control group. The meta-analysis showed that patients treated with G-CSF had a significantly lower risk of sepsis compared to those treated with standard medical therapy (SMT) alone (RR = 0.53, 95% CI: 0.35–0.80, P<0.05, I^2^ = 0%).

Three studies involving 205 ACLF patients reported hepatic encephalopathy (HE), with 103 patients in the G-CSF treatment group and 102 in the control group. The meta-analysis indicated that there was no significant difference in the risk of HE between patients treated with G-CSF and those treated with SMT alone (RR = 0.64, 95% CI: 0.27–1.50, P = 0.30, I^2^ = 0%).

Bleeding was reported in 8 studies involving 461 ACLF patients, 231 were in the G-CSF treatment group and 230 were in the control group. The meta-analysis showed that compared with SMT, patients receiving G-CSF did not have a significantly reduced risk of bleeding (RR = 1.04, 95% CI: 0.58–1.87, P = 0.90, I^2^ = 0%).

Five studies involving 283 ACLF patients reported Hepatorenal syndrome (HRS), with 142 patients in the G-CSF treatment group and 141 in the control group. The meta-analysis indicated that there was no significant difference in the risk of HRS between patients treated with G-CSF and those treated with SMT alone (RR = 0.58, 95% CI: 0.31–1.07, P = 0.08, I^2^ = 0%). (S1 Fig).

### Sensitivity analysis

We performed a sensitivity analysis in which one study was removed at a time and the pooled estimates were recalculated for the remaining studies. The results showed that the overall findings were not substantially influenced by any individual study (S2 Fig).

### Publication bias

Asymmetry was observed in the visual inspection of the funnel plot, indicating the possibility of publication bias. Therefore, sensitivity analysis was conducted using the Egger’s test and trim and fill method [24]. The difference between the original effect size estimate and the corrected effect size estimate was not substantial, indicating that publication bias did not have a significant impact on the results (S1 Table).

## Discussion

Acute-on-chronic liver failure (ACLF) is a severe and potentially fatal condition that occurs in patients with pre-existing chronic liver disease. It is characterized by acute decompensation and organ failure, which significantly increases morbidity and mortality rates. The treatment of ACLF remains a major challenge, and current therapeutic options are limited. In recent years, granulocyte colony-stimulating factor (G-CSF) has been proposed as a potential therapy for ACLF. G-CSF is a cytokine that stimulates the production and differentiation of neutrophils and has been shown to have immunomodulatory and anti-inflammatory effects [25]. In this meta-analysis, we evaluated the efficacy and safety of G-CSF therapy in the treatment of ACLF.

According to our study, the Child-Turcotte-Pugh (CTP) and Model for End-Stage Liver Disease (MELD) scores were utilized to predict disease severity and mortality at 3 months among patients diagnosed with end-stage liver disease [26]. Our results indicate that treatment with G-CSF was significantly associated with improved liver function, as evidenced by improvements in both MELD and CTP scores. This improvement was observed after only 7 days of treatment and persisted for up to 30 days post-treatment. Despite these positive findings, there were no significant differences observed in serum albumin and total bilirubin levels between the G-CSF and control groups. However, there was a slight improvement in International Normalized Ratio (INR) levels within one month of treatment. These results suggest that G-CSF therapy may have a beneficial effect on liver function in patients with ACLF. Recently, several definitions and prognostic scores have been developed specifically for ACLF. In particular, the CLIF-C ACLF score and the COSH-ACLF score provide higher predictive performance for patients with ACLF. However, these scores were rarely used in the articles included so far. It is expected that more studies will use prognostic scoring systems with higher sensitivity and specificity, which will lead to more valuable results.

Our analysis investigated the development of complications and causes of death in patients with ACLF, a serious condition with high mortality rates [27]. We found that G-CSF therapy, compared to standard medical therapy, was associated with a lower risk of sepsis in these patients. G-CSF is able to inhibit the activation of Kupffer cells by lipopolysaccharide (LPS) and significantly attenuate LPS-induced inflammatory response, decreasing the elevation of the pro-inflammatory factor tumor necrosis factor-alpha (TNF-alpha) and increasing the activity of the inflammation inhibitor interleukin-6 (IL-6). Sarin SK et al. [28] reported a significant increase in peripheral blood and intrahepatic dendritic cells and a decrease in gamma-interferon (IFN-γ)-secreting CD8+ T cells in patients with ACLF treated with G-CSF. Myeloid dendritic cells are a key part of the host’s anti-microbial response, and administration of G-CSF increases circulating and intrahepatic myeloid dendritic cells, contributing to the achievement of immunomodulation. The decrease in IFN-γ also played an important role in reducing liver injury. This immunomodulatory effect of G-CSF promotes healing and functional recovery of liver tissue and reduces the number of patients who develop serious complications such as sepsis. These findings appear to be compatible with our results. However, we did not find any significant differences in the risk of hepatic encephalopathy, bleeding, or hepatorenal syndrome between the G-CSF and control groups. These findings suggest that G-CSF therapy may have a protective effect against infections, which are a major challenge in the treatment of ACLF. ACLF is characterized by marked pathophysiological features, including immune dysfunction and susceptibility to infections [29,30]. G-CSF is an immunomodulatory glycoprotein that exerts anti-inflammatory and immunomodulatory effects, which may help reduce the occurrence of bacteremia and infections, particularly in patients with ACLF [25].

Our meta-analysis also demonstrated that G-CSF therapy was associated with a significant improvement in survival rates in ACLF patients, with reduced risks of death at 30, 60, and 90 days. Moreover, both doses of G-CSF (5 μg/kg/day and 10 μg/kg/day) improved survival rates, suggesting that G-CSF may be effective at different doses.

Several studies have demonstrated the safety and efficacy of G-CSF in mobilizing bone marrow stem cells and improving the clinical, biochemical, and histological status of patients with end-stage liver disease (ESLD) [31,32]. G-CSF can stimulate the bone marrow to release stem cells (CD34+), which can migrate to the liver and differentiate into mature hepatocytes. Additionally, G-CSF can reduce the production of interferon-gamma, improve the local microenvironment of the liver, promote liver repair, and improve liver injury, resulting in improved liver function, reduced risk of complications, decreased risk of infections, and improved survival [33]. For example, Spahr et al. [34] found that G-CSF administration in ACLF patients improved liver function by increasing the number of CD34+ cells in the peripheral blood and promoting the proliferation of hematopoietic stem cells in liver tissue. Duan et al. [10] and Garg et al. [11] further suggested that G-CSF application could effectively improve the prognosis of patients with ACLF by promoting hepatocyte regeneration through mobilization of CD34+ cells in vivo. Taken together, our findings suggest that G-CSF therapy has potential therapeutic benefits for patients with ACLF, especially those at high risk of infections, and warrants further investigation in clinical trials.

The pathogenic factors of ACLF are complicated, and the morbidity and mortality rates are still as high as 50%-90% under comprehensive internal medicine treatment. Because of the different etiologic and pathogenic factors of ACLF between the East and the West, there are many controversies between scholars from both sides on the definition, diagnostic criteria, pathogenesis and other aspects of ACLF. Currently, the definition and diagnostic criteria of ACLF commonly used in the international arena include European Association for the Study of the Liver-Chronic Liver Failure (EASL-CLIF), Chinese Group on the Study of Severe Hepatitis criteria (COSSH-ACLF), Asian Pacific Association for the Study of the Liver-ACLF (APASL-ACLF), and North American Consortium for the Study of End-stage Liver Disease (NACSELD) criteria. Controversies about the type of acute injury induced (intrahepatic or extrahepatic factors), the stage of chronic liver disease (with or without cirrhosis), and whether to include extrahepatic organ failure are the main reasons for the lack of uniformity in the diagnostic criteria for ACLF worldwide. Engelmann et al [18] conducted a multicenter study with European subjects and found no significant benefit of G-CSF in ACLF patients. Whether this result is related to potential ethnic differences and non-uniform diagnostic criteria remains to be further investigated. A global, multicenter, prospective study is urgently needed to establish uniform diagnostic criteria and prognostic scores for ACLF.

The present study has several limitations that should be noted. Firstly, the majority of the studies included in this analysis were conducted in Asian countries, which may limit the generalizability of the findings to other populations. Secondly, the total sample size is relatively small, which may affect the reliability and clinical relevance of the analysis results. Lastly, heterogeneity was observed in the results, which may be due to differences in study populations, diagnostic criteria, G-CSF dosage and treatment duration across trials, as well as limited data availability for some studies, resulting in a relatively weak level of evidence. Therefore, additional randomized, double-blind controlled trials with larger sample sizes and more standardized protocols are needed to confirm the efficacy and safety of G-CSF in the treatment of ESLD.

In conclusion, our meta-analysis indicates that G-CSF therapy is associated with significant improvements in liver function and survival rates in ACLF patients. Our findings suggest that G-CSF may be a promising therapy for ACLF and warrants further investigation. However, the potential risks and benefits of G-CSF therapy should be carefully weighed before implementation in clinical practice. Future studies should focus on identifying the optimal dose and duration of G-CSF therapy and determining the long-term safety and efficacy of this treatment.

## Supporting information

S1 Checklist PRISMA NMA checklist of Items to include when reporting a systematic review involving a network meta-analysis. (DOCX)

S1 Fig a. Analysis of development of complications. Sepsis. b. Analysis of development of complications. Hepatic encephalopathy. c. Analysis of development of complications. Gastrointestinal bleeding. d. Analysis of development of complications. Hepatorenal syndrome.
(TIF)

S2 Fig a. Sensitivity analysis of 30D. b. Sensitivity analysis of 60D. c. Sensitivity analysis of 90D.
(TIF)

S1 Table The overall effect sizes before/after applying the trim-and-fill methods. (DOCX)

## Abbreviations

G-CSF: granulocyte-colony stimulating factor
ACLF: acute-on-chronic liver failure
BMSCs: bone marrow stem cells
MELD: Model for end stage of liver disease
CTP-scores: Child–Turcotte–Pugh scores
INR: international normalized ratio
HE: hepatic encephalopathy
RCT: randomized clinical trials
RR: risk ratios
Cl: confidence intervals
MD: mean differences
SD: standard deviation
