# Supplementary figures and images for: The efficacy and safety of granulocyte colony-stimulating factor in the treatment of acute-on-chronic liver failure: A systematic review and meta-analysis
Source: PLoS One. 2023 Nov 30;18(11):e0294818. doi: 10.1371/journal.pone.0294818 (PMC10688871; doi:10.1371/journal.pone.0294818)

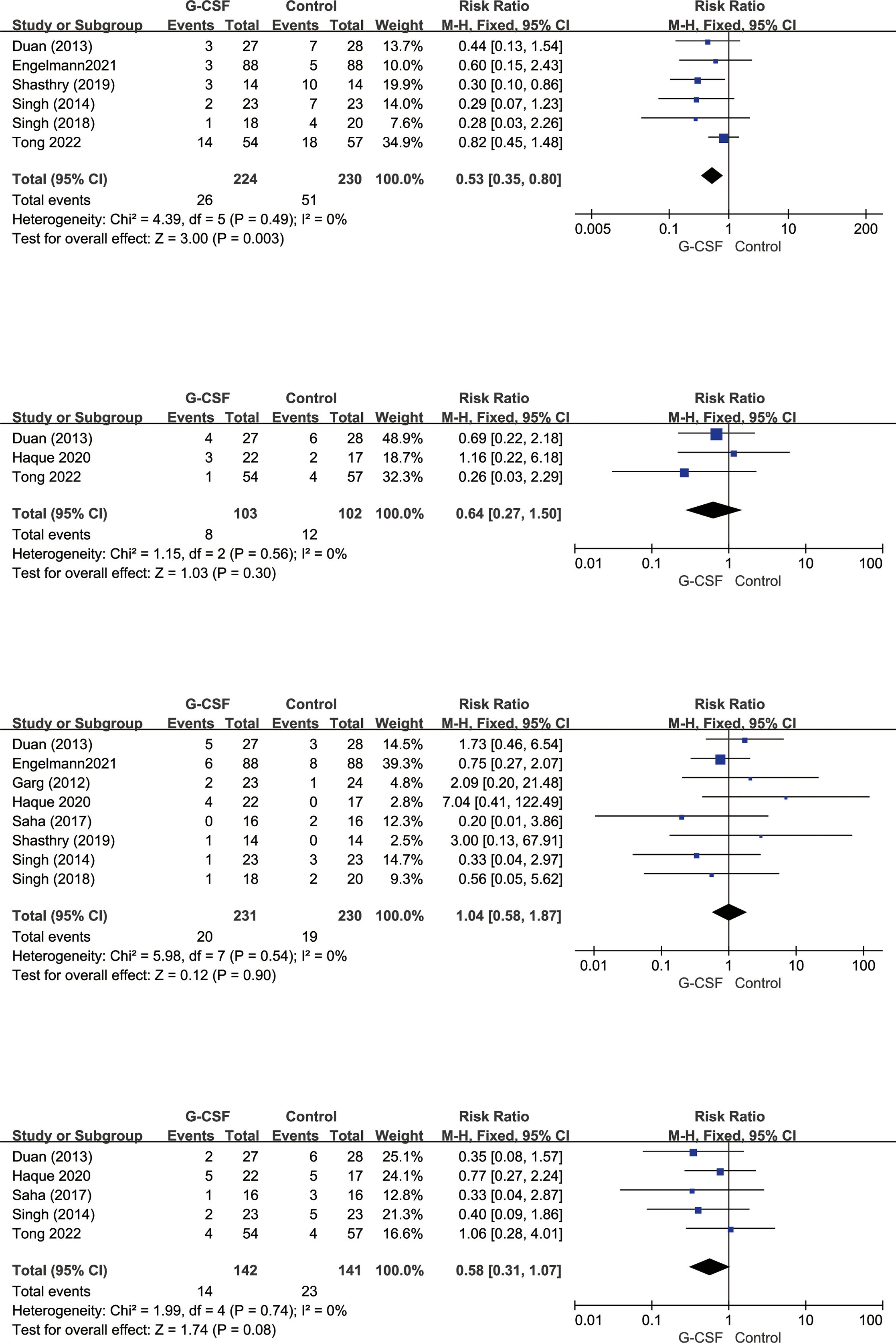

Supplement: S1 Fig — a. Analysis of development of complications. Sepsis. b. Analysis of development of complications. Hepatic encephalopathy. c. Analysis of development of complications. Gastrointestinal bleeding. d. Analysis of development of complications. Hepatorenal syndrome. (TIF) [file pone.0294818.s002.tif]

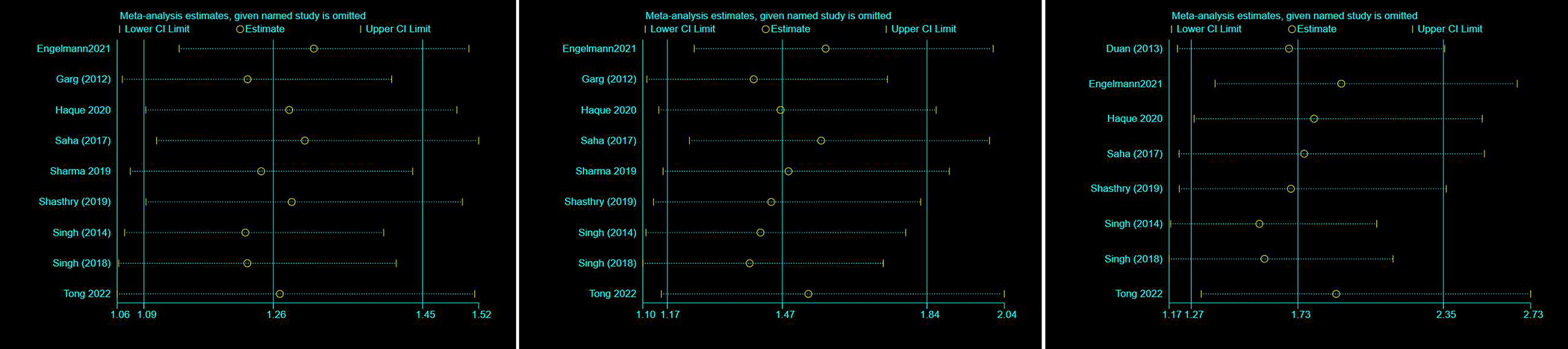

Supplement: S2 Fig — a. Sensitivity analysis of 30D. b. Sensitivity analysis of 60D. c. Sensitivity analysis of 90D. (TIF) [file pone.0294818.s003.tif]
