# Supplementary material for: The efficacy and safety of granulocyte colony-stimulating factor in the treatment of acute-on-chronic liver failure: A systematic review and meta-analysis
Source: PLoS One. 2023 Nov 30;18(11):e0294818. doi: 10.1371/journal.pone.0294818 (PMC10688871; doi:10.1371/journal.pone.0294818)
Supplement: S1 Table — (DOCX) [file pone.0294818.s004.docx]

**Supplementary Table 1. The overall effect sizes Before/After applying the trim-and-fill methods**

|  | **Result of Egger's test** | **Imputed missing studies** | **Before trim-and-fill methods** | **After trim-and-fill methods** |
| --- | --- | --- | --- | --- |
| 30-DAY survival | P =0.07 | 0 | -0.86 (-1.28 to -0.45) | -0.86 (-1.28 to -0.45) |
| 60-DAY survival | P =0.03 | 1 | -0.98 (-1.33 to -0.62) | -0.95 (-1.31 to -0.60) |
| 90-DAY survival | P=0.01 | 5 | 0.07 (0.03-0.12) | 0.06 (0.01-0.10) |
| CTP scores (7-DAY) | P=0.99 | 0 | -0.51 (-0.91 to -0.11) | -0.51 (-0.91 to -0.11) |
| CTP scores (30-DAY) | P=0.32 | 2 | -0.36(-0.69 to 0.03) | -0.55(-0.83 to -0.27) |
| MELD scores (7-DAY) | P=0.37 | 1 | -0.39 (-0.70 to -0.07) | -0.56 (-0.84 to -0.28) |
| MELD scores (30-DAY) | P=0.43 | 0 | -0.40 (-0.80 to 0.01) | -0.40 (-0.80 to 0.01) |
| Sepsis | P=0.045 | 0 | -0.90 (-1.27 to -0.53) | -0.90 (-1.27 to -0.53) |
